# Is Metformin the Answer for Distressed Females with Menstrual Irregularities?

**DOI:** 10.7759/cureus.5460

**Published:** 2019-08-22

**Authors:** Chavi Tejpal, Ishan Poudel, Nusrat Jahan

**Affiliations:** 1 Family Medicine, Department of Research, California Institute of Behavioral Neurosciences and Psychology, Fairfield, USA; 2 Internal Medicine, Department of Research, California Institute of Behavioral Neurosciences and Psychology, Fairfield, USA

**Keywords:** polycystic ovary syndrome, metformin, menstrual irregularities

## Abstract

This literature review is aimed to determine if metformin alone improves menstrual irregularities in females with the polycystic ovarian syndrome. The current literature review involves females with polycystic ovarian syndrome experiencing menstrual irregularity. The data was collected in PubMed and inclusion criteria included articles published in the past 10 years, articles involving only humans, articles written in the English language and considering women age 19 or more. The number of discovered articles was 1550 after the first search and only 25 articles that met the inclusion criteria were selected after quality assessment. The selected 25 articles that met the inclusion criteria after a review showed evidence for regulating menstrual cycles with metformin therapy alone among females with the polycystic ovarian syndrome. When metformin was used in addition to other pharmacologic agents, there was a more significant restoration of menstrual cycles. Monotherapy with metformin is found to be highly effective in treating menstrual irregularities experienced among patients with the polycystic ovarian syndrome. Greater improvement was noted with the addition of another agent to metformin.

## Introduction and background

The polycystic ovarian syndrome affects women of childbearing age and is a clinical diagnosis. The etiology of the polycystic ovarian syndrome is unknown but may be attributed to insulin resistance. Patients frequently present with symptoms such as menstrual irregularities, androgen excess, and polycystic ovaries. While androgen excess often presents with acne and hirsutism, menstrual irregularities often lead to infertility. Treating menstrual irregularities is a crucial component to the polycystic ovarian syndrome. Menstrual irregularities caused by polycystic ovaries leads to infertility, resulting in distress among women trying to conceive [1-3]. 

Hormonal pharmacologic therapies such as oral contraceptives have been used for the treatment of menstrual irregularities in polycystic ovarian syndrome. In addition to hormone therapy, metformin was used to treat irregular menses in females with a history of oligo-menorrhea or amenorrhea. The exact mechanism of metformin in regulating menstrual cycle is still unknown. However, it may be attributed to insulin sensitization thereby targeting the insulin resistance caused by androgen excess [1-3]. This literature review is aimed to determine the effectiveness of metformin in regulating menstrual cycles among females with the polycystic ovarian syndrome.

## Review

Method 

The data was collected from PubMed using regular keywords “polycystic ovary” and “metformin.” The following inclusion criteria were applied in the following order - literature published within 10 years, articles involving only human subjects, articles published only in English language and the age of the subjects 19 years or more. The collection of the articles for the study was done ethically.

Results

After using regular keywords on PubMed, a total of 1550 articles were identified. Among the 1550 articles, 347 were obtained with the application of filters. Table 1 shows the total number of articles obtained in order after applying inclusion criteria in PubMed.

**Table 1:** Keywords search after applying inclusion criteria in PubMed

Articles	Results
Total articles found	1550
After inclusion criteria	
10 years	890
Humans	706
English language	658
Age 19 or more	347

Of the 347 articles obtained, each article was individually reviewed based on the abstract content. Three hundred and twenty-two articles were not selected for review due to one or more of the following reasons:

- lack of disease of interest;

- lack of menstrual irregularities;

- lack of metformin use for menstrual irregularity;

- studies with no possible derivation of information with quality assessment.

The number of articles obtained after removal of 322 articles was 25, among which 16 were free abstracts available online for review and nine were free with full text available online for review. Of the articles included in the data, there were 21 randomized controlled trials, two literature reviews, and one systemic review.

In addition to metformin, other drugs used as comparators in the randomized clinical trials were clomiphene citrate, oral contraceptives, ethinylestradiol, desogestrel, simvastatin, rosiglitazone, orlistat, n-acetylcysteine, letrozole, calcium & vitamin D, pioglitazone, saxagliptin, spironolactone, flutamide, lipoic acid, monacolin K and myo-inositol. Non-pharmacologic therapies used in clinical trials were lifestyle modifications such as caloric restriction and exercise [3-12]. 

The 25 selected articles showed evidence for metformin therapy to be efficacious in regulating menstrual cycles of females with the polycystic ovarian syndrome. When metformin was used with other pharmacologic agents, there was a recovery of menstrual cycles as well. Of the articles collected, the randomized clinical trials involving metformin showed improvement in menstrual cycle irregularity with metformin [3, 10-17]. Table 2 shows the analysis of various parameters seen in the randomized clinical trials.

**Table 2:** Analysis of various parameters mg - milligram; LH - luteinizing hormone; FSH - follicle-stimulating hormone; BID - two times a day; BMI - body mass index; COC - combined oral contraceptives; N/A - not available; TG - triglyceride; HDL - high-density lipoprotein

Randomized Controlled Trial									
Author/Year	Population	Metformin (dose in mg if applicable)	Arms	Length of Study (Months)	Results				
					Menses	LH/FSH	Weight	Testosterone	Other
Seyam et al. 2018 [16]	65	500 mg three times daily	Group A simvastatin + metformin, group B simvastatin, group C metformin	12	Improved	Decrease serum LH level	Decrease BMI	Decrease	Decrease of total cholesterol, low-density lipoprotein, triglycerides, increase in high-density lipoprotein decreased hirsutism, acne, ovarian volume
Alpañés et al. 2017 [3]	22	850 mg BID	Group with combined oral contraceptive (COC) plus spironolactone versus metformin	12	Menstrual dysfunction was less frequent with COC plus spironolactone	N/A	N/A	Total testosterone, free testosterone	COC + spironolactone showed more decrease in hirsutism, androstenedione, dehydroepiandrosterone sulphate
Tagliaferri et al. 2017 [11]	34	850 mg BID	Group metformin versus myoinositol	6	Improved menstrual pattern with metformin	Metformin showed LH significant decreased	Metformin showed a decrease in weight	N/A	Metformin showed a decrease in estradiol levels and androgens
Elkind-Hirsch et al. 2017 [17]	3	2,000 mg	Combined saxagliptin 2,000 mg-metformin 5 mg, saxagliptin 5 mg, or metformin 2,000 mg	4	Saxagliptin-metformin greatly improved the menstrual irregularity	N/A	BMI, waist/height ratio and waist circumference decreased in all groups	N/A	Significant decrease in triglyceride, TG/HDL cholesterol ratio, and blood glucose level in the saxagliptin-metformin and saxagliptin groups only
Mazza et al. 2014 [12]	28	1700 mg	Group A metformin (1700 mg/day), group B with metformin (1700 mg/day) + spironolactone (25 mg/day)	N/A	Menses improved more in metformin 1700 mg a day	N/A	N/A	Significant decrease in testosterone in both groups	Significant decrease in group A and B of androstenedione and hirsutism
Firouzabadi et al. 2012 [13]	50	1500 mg/day	Group I metformin 1500 mg a day, group II metformin 1500 mg a day + calcium 1000 mg a day + vitamin D 100000 IU per month	6	Menses improved more in metformin 1500 mg a day	N/A	Significant decrease in BMI in group II	N/A	Follicle maturation, and infertility improved group II > group I

Irrespective of the dosage, it is observed that even at a 500 mg dosage, metformin therapy showed improvement in regulating menstrual cycles. In addition, metformin also showed a decrease of hormone levels such as luteinizing hormone (LH), luteinizing hormone/follicle-stimulating hormone (LH/FSH) ratio, testosterone, estradiol, dehydroepiandrosterone sulfate (DHEA-S) and progesterone. Furthermore, metformin was effective in weight reduction and hirsutism [15, 18-19].

Table 3 below illustrates the effects seen with metformin monotherapy at various doses in the following randomized clinical trials in patients with polycystic ovarian syndrome.

**Table 3:** Randomized clinical trials involving monotherapy of metformin at various doses mg - milligram; LH - luteinizing hormone; FSH - follicle-stimulating hormone; BID - two times a day; TID - three times a day; BMI - body mass index; CRP - c-reactive protein; DHEA-S - dehydroepiandrosterone sulfate; N/A - not available

Randomized Control Trial with metformin only								
Author/ Year	Population	Dose (mg)	Length of Study (Months)	Results				
				Menses	LH/FSH	Weight	Testosterone	Other
Fulghesu et al. 2012 [15]	201	500 mg BID, 500 mg TID, 850 mg BID	6	Increase of more than two menstrual cycles/year	N/A	N/A	Greater reduction in plasma testosterone levels who had a higher basal BMI	N/A
Yang et al. 2018 [18]	119	The first month 500 mg once; the second month 1000 mg once; third month & onwards 1500 mg once	24	Increased frequency	Decreased LH	Decreased BMI	Decreased	N/A
Velija-Ašimi et al. 2013 [19]	100	1000-1500 mg BID/TID	12	Restored	Decreased LH, LH/FSH	Reduced weight, waist circumference, BMI	Decreased	Decrease estradiol, CRP, DHEA-S and progesterone, hirsutism

## Conclusions

The objective of the current literature review is to determine the effectiveness of metformin in regulating menstrual cycles among women diagnosed with the polycystic ovarian syndrome experiencing irregular menses. The use of metformin has demonstrated an improvement in regulating menses significantly with a greater improvement when metformin is used as an adjunctive to another pharmacologic agent. It is to be determined whether these patients will require lifelong metformin therapy or if insulin sensitization can be achieved at a specific number of doses required thereby leaving room for speculation if metformin can be halted after a certain number of doses. One wonders if metformin’s effect at regulating menses is tied with insulin sensitization and if serum testosterone plays a role. 

## References

[1] Al Khalifah RA, Florez ID, Dennis B, Thabane L, Bassilious E (2016). Metformin or oral contraceptives for adolescents with polycystic ovarian syndrome: a meta-analysis. J Pediatr.

[2] Legro RS, Arslanian SA (2013). Diagnosis and treatment of polycystic ovary syndrome: an endocrine society clinical practice guideline. J Clin Endocrinol Metab.

[3] Alpañés M, Álvarez-Blasco F, Fernández-Durán E, Luque-Ramírez M, Escobar-Morreale HF (2017). Combined oral contraceptives plus spironolactone compared with metformin in women with polycystic ovary syndrome: a one-year randomized clinical trial. Eur J Endocrinol.

[4] Elkind-Hirsch KE, Paterson MS, Seidemann EL, Gutowski HC (Issue 1). Short-term therapy with combination dipeptidyl peptidase-4 inhibitor saxagliptin/metformin extended release (XR) is superior to saxagliptin or metformin XR monotherapy in prediabetic women with polycystic ovary syndrome: a single-blind, randomized, pilot study. Fertil Steril.

[5] Fruzzetti F, Perini D, Russo M, Bucci F, Gadducci A (2017). Comparison of two insulin sensitizers, metformin and myo-inositol, in women with polycystic ovary syndrome. Gynecol Endocrinol.

[6] Seyam E, Hefzy E (2018). Long-term effects of combined simvastatin and metformin treatment on the clinical abnormalities and ovulation dysfunction in single young women with polycystic ovary syndrome. Gynecol Endocrinol.

[7] Altinok ML, Ravn P, Andersen M, Glintborg D (2018). Effect of 12-month treatment with metformin and/or oral contraceptives on health-related quality of life in polycystic ovary syndrome. Gynecol Endocrinol.

[8] Yang P-K, Hsu C-Y, Chen M-J (2018). The efficacy of 24-month metformin for improving menses, hormones, and metabolic profiles in polycystic ovary syndrome. J Clin Endocrinol Metab.

[9] Kazerooni T, Shojaei-Baghini A, Dehbashi S, Asadi N, Ghaffarpasand F, Kazeroonic Y (2010). Effects of metformin plus simvastatin on polycystic ovary syndrome: a prospective, randomized, double-blind, placebo-controlled study. Fertil Steril.

[10] Figurová J, Dravecká I, Petríková J, Javorský M, Lazúrová I (2017). The effect of alfacalcidiol and metformin on metabolic disturbances in women with polycystic ovary syndrome. Horm Mol Biol Clin Investig.

[11] Tagliaferri V, Romualdi D, Immediata V (2017). Metformin vs myoinositol: which is better in obese polycystic ovary syndrome patients? A randomized controlled crossover study. Clin Endocrinol.

[12] Mazza A, Fruci B, Guzzi P (2014). In PCOS patients the addition of low-dose spironolactone induces a more marked reduction of clinical and biochemical hyperandrogenism than metformin alone. Nutr Metab Cardiovasc Dis.

[13] Firouzabadi RD, Aflatoonian A, Modarresi S, Sekhavat L, Taheri SM (2012). Therapeutic effects of calcium & vitamin D supplementation in women with PCOS. Complement Ther Clin Pract.

[14] Oner G, Muderris II (2011). Clinical, endocrine and metabolic effects of metformin vs N-acetyl-cysteine in women with polycystic ovary syndrome. Eur J Obstet Gynecol Reprod Biol.

[15] Fulghesu AM, Romualdi D, Di Florio C (2012). Is there a dose-response relationship of metformin treatment in patients with polycystic ovary syndrome? Results from a multicentric study. Hum Reprod.

[16] Seyam E, Hefzy E (2018). Long-term effects of combined simvastatin and metformin treatment on the clinical abnormalities and ovulation dysfunction in single young women with polycystic ovary syndrome. Gynecol Endocrinol.

[17] Elkind-Hirsch KE, Paterson MS, Seidemann EL, Gutowski HC (2017). Short-term therapy with combination dipeptidyl peptidase-4 inhibitor saxagliptin/metformin extended release (XR) is superior to saxagliptin or metformin XR monotherapy in prediabetic women with polycystic ovary syndrome: a single-blind, randomized, pilot study. Fertil Steril.

[18] Yang PK, Hsu CY, Chen MJ (2018). The efficacy of 24-month metformin for improving menses, hormones, and metabolic profiles in polycystic ovary syndrome. J Clin Endocrinol Metab.

[19] Velija-Ašimi Z (2013). Evaluation of endocrine changes in women with the polycystic ovary syndrome during metformin treatment. Bosn J Basic Med Sci.

